# State of the Patients of the Bristol Dispensary, from the 1st of July to the 1st of August, 1801

**Published:** 1801-09

**Authors:** 


					State of Diseases in London* igj*
State of the Patients of the Bristol Dispensary, from the ift ef
July tt the if of Augujl, 1801.
Typhus Fevers - 39
Putrid Fevers - - 2
Inflammatory Fevers - 6
Bilious Fevers - - - - 3
Miliary Fever - - - - 1
Intermittent Fever - - - 1
An a ?1 rest - - - - 2
Afcites - -- --- - 1
Pleurify - - - - - - 1
Haemorrhoids - - - ? 1
Variola * ----- l
Podagra ------ 2
Confumption - - - - 5
Ophthalmia ----- i
Gaftrodynia - - f
Dyfpepfia ----- j
Diarrhoea - - - - - I
Afthma - - - - -- z
Eryfipelas ----- 3
Sarcocele ----- i
Scorbutic - - - - x
Tenefmus - - - - - x
Exanthema ----- i
Dyfenteria - - - - 2
Ilheymatifm - - 1
Cardialgia - - - - - 1
Uterine Haemorrhage - - 2
Sctophula ----- i

				

